# Experiences of felt presence in first episode psychosis

**DOI:** 10.1038/s41537-025-00690-2

**Published:** 2025-11-26

**Authors:** Ben Alderson Day, Peter Moseley, Angela Woods, Guy Dodgson, Stephanie Common, Charles Fernyhough

**Affiliations:** 1 Department of Psychology, Durham University, Durham, UK; 2 Department of Psychology, Northumbria University, Durham, UK; 3 Institute for Medical Humanities, Durham University, Durham, UK; 4 Cumbria, Northumberland, Tyne & Wear NHS Foundation Trust, Newcastle upon Tyne, UK; 5 Tees, Esk, & Wear Valleys NHS Foundation Trust, Darlington, UK

**Keywords:** Psychosis, Schizophrenia, Human behaviour

## Abstract

Felt presence (FP) – sensing another person without clear sensory evidence – has been described in psychosis for over a century but rarely studied due to challenges in recognition and assessment. Recently FP has been identified as a transdiagnostic phenomenon and highlighted by people with lived experience of psychosis as a clinical priority. Here we describe FP presentation in a first-episode psychosis sample and report preliminary associations with affect, gender, and psychopathology.

## Introduction

Since Jaspers^1^, felt presence (FP) has been a documented but understudied aspect of the unusual and challenging phenomenology of psychosis. Studies of FP have focused predominantly on neurological rather than psychiatric disorders, especially Parkinson’s^2^. This picture is changing, however, with lived experience scholars calling for greater attention to FP^3^ and growing awareness of the role of the bodily self in schizophrenia^4^.

Although sometimes classified as extracampine hallucinations^5^ (i.e. impossible sensations outside of the sensory field^6^), FP typically involves a lack of sensory content with the sensation being described as one of “just” feeling a person is there^7^. This makes assessment particularly challenging with a double risk of presences being endorsed simply as secondary interpretations of other hallucinations (e.g. “the voices make me feel like someone is always there”) or feelings of paranoia (“I think that someone is watching me”). Identifying FP as a distinct experience therefore requires careful phenomenological assessment to understand its clinical importance beyond other aspects of psychopathology more readily recognised.

From 2018 to 2020, Durham University’s *Hearing the Voice* (HtV) conducted phenomenological interviews with 40 voice-hearers using Early Intervention in Psychosis services for the first time. Designed by an interdisciplinary team, the HtV Phenomenology Interview explored several aspects of the experience of hearing voices including FP, reported by 52% of participants^8^. Here, we present some detailed examples of FP in this group, and secondary analyses of other aspects of psychopathology associated with the phenomenon in early psychosis.

As shown in Table 1, accounts of FP often have spatial and somatic qualities to them, in which people are aware of the presence being close by or alongside, and may have bodily sensations (such as chills). This was described by Jaspers as *leibhaftige Bewusstheit* or “physical awareness”^1^, which he considered a primary experience separable from other features of psychosis^9^. Phenomenological coding of the HtV interviews allowed us to explore whether having FP made people more likely to also have other sensory experiences. Those reporting FP were more likely to experience tactile hallucinations (see Table 2) and to mentally link presences with sensations in other modalities, denoting a singular multisensory agent. Importantly this was largely not experienced simultaneously, as indicated by multiple participants reporting the presence of “voices” even when there were not speaking. Because of their resemblance to depersonalisation – as seen in some neurological cases^10^ – FP may be expected to be associated with dissociation and changes to bodily states: this was also evident in the sample, albeit to a lesser degree than tactile or multisensory phenomena. The association with tactile experiences in particular is consistent with recent findings in a large general population study of hallucinatory experiences^11^.

**Table 1:** Descriptions of felt presence in psychosis at baseline (Y1), 12-month (Y2) and 24-month assessment (Y3).

Alex (Y1)	Sometimes yes, because like I say, you’re that used to hearing them and comment on what you’re doing, when they’re quiet you just think they’re sitting, watching, waiting to comment.
Bill (Y1)	At the moment it’s more of a presence. Because it has no figure and no shape and no form you don’t know what it is yet, so you can only feel the experience as a presence, you know.
Gail (Y1)	I feel like there’s constantly someone watching me.
Kate (Y1)	Ah, ah, you know like when someone’s lying behind you and it’s just like a dead weight
Dawn (Y1)	I constantly felt like she’s in the room with me, or with whoever, or … and I felt very detached from what was going on. So say we’re sat here, I’d feel like you … you were having the conversation and I’m like a ghost almost, and she’s with me, if … does … if that makes sense.
Emma (Y1)	You know that feeling you get when you’ve, when like there’s another person there? Do you know what I mean? If you go in an empty house and there’s someone in there that … I don’t know, it’s hard to … like that static-y feeling, you know that, as if someone is there.
Iris (Y1)	I know he’s always there. You can just sense it sometimes, just sense that he’s there. Like even talking to you, I know that he’s there, he’s just … he knows that … I just know that he’s wanting to say something
Liam (Y1)	Yeah, it feels like they’re with me, it’s, I just feel the presence of someone with me all the time, watching me.
Kath (Y2)	I do often feel like there’s people around us, and I do often feel like there’s people … like a lot … like I’m scared of mirrors, like I hate … like if I’m in bed, like I can’t have a mirror like in any way where I can see it because … I don’t know like I feel like if there’s a mirror there, people can … people will be looking at us, and like if they’re not already, they’re gonna if there’s a mirror there. Like so I don’t feel like the voices are there because I know that they’re not real but I do often feel like something’s there, something’s watching like … and I wouldn’t say it’s ghosts, because again like I don’t think ghosts are real, but that’s as close as I can describe it.
Will (Y3)	They won’t have made a noise but I can sense that they’re there, I can feel like … I can feel like the presence of them’s there. I guess the best way to describe it probably is for me is you know when you walk into a situation and you get that instant shiver of where you feel like … and then you can feel like your arms … the hairs on your arms stand up and you get like that goosebumpy feeling? That’s the feeling like I get when I can sense that they’re there. It’s like that, I feel like as if that someone’s … you know that you say you get that feeling like someone’s walked over your grave, you get that … (gasps) you know that feeling through your body, that’s the best way to describe it.
Xander (Y3)	I came home one day and I heard someone like … sorta shouting for help, but no one was in the house. Like the only two people were in the house were my mam and sister which were in … they were in the same room as me, it wasn’t them. And it like had the same … sort of feeling, I remember the same feeling that I sort of get when I hear my voices, like … even if my voices aren’t talking, I can sort of sense that they’re there. If that makes sense? And I remember the feeling of the same sort of sense. How do I describe it? Have you ever felt like someone’s like watching you, or felt like someone’s like very close behind ya? It’s kinda just like that. It’s like that feeling that someone’s close by…. I think I get like a sort of … like a gut feeling, like a weird feeling in my stomach sometimes.

**Table 2:** Comparing demographics, psychopathology and code associations for felt presence.

		Felt presence (*N* = 21)	No felt presence (*N* = 19)	
		*M (SD)*		*d (95% CI)*
Age		25.95 (7.38)	31.74 (11.65)	0.6 (−0.03;1.23)
Voice Onset^a^		19.10 (10.90)	26.08 (15.15)	0.53 (−0.11;1.16)
PSYRATS-AH		28.00 (4.70)	23.32 (6.98)	0.80 (0.14;1.44)
- Physical		9.48 (2.32)	9.16 (2.12)	0.14 (−0.48; 0.76)
- Emotional		11.48 (3.19)	8.26 (4.43)	0.84 (0.19; 1.48)
- Cognitive		7.05 (1.36)	5.89 (2.33)	0.61 (−0.02; 1.24)
PSYRATS-D		7.52 (8.52)	6.05 (6.77)	0.19 (−0.43; 0.81)
				lgOR (95% CI)
Gender	F	71%	29%	−1.32 (−2.66; 0.02)
	M	39%	61%	
Tactile	Y	89%	11%	2.40 (0.21; 4.60)
	N	42%	58%	
Bodily States	Y	58%	42%	0.60 (−0.72; 1.91)
	N	43%	57%	
Multisensory	Y	73%	27%	1.19 (−0.33; 2.70)
	N	45%	55%	
Dissociation	Y	67%	33%	0.84 (−0.58; 2.25)
	N	46%	54%	
Fear	Y	71%	29%	1.99 (0.55; 3.42)
	N	25%	75%	
Anxiety	Y	54%	46%	0.15 (−1.15; 1.45)
	N	50%	50%	
Depression	Y	52%	48%	−0.01 (−1.25; 1.23)
	N	53%	47%	
Paranoia	Y	55%	45%	0.20 (−1.04; 1.44)
	N	50%	50%	
Direct Address	Y	61%	39%	2.22 (−0.01; 4.45)
	N	14%	86%	

^a^Analysis in *n* = 39 as one participant could not estimate voice onset age.

FP in psychosis are often described as particularly unsettling, in a similar manner to those reported during sleep paralysis^12^. Accordingly, FP in the HtV data were notably associated with feelings of fear, but not with other feelings like anxiety, depression or paranoia (i.e. specific suspicion of others). The sense of presences specifically targeting and paying attention to the perceiver was common in participant descriptions, and linked to voices that directly addressed the listener. In addition, there was also some evidence that FP were more likely to be reported by female participants, by younger participants, and those with an earlier onset of hearing voices.

We explored the question of whether FP are associated more with hallucinations or delusions by comparing PSYRATS^13^ scores for those with and without FP. Participants reporting presences scored higher on overall severity of auditory hallucinations (AH) but participants with FP scored no higher on delusion severity. Dividing AH scores up into subscales indicated that PSYRATS emotion scores were higher in the FP group (compared to those without FP), followed by cognitive scores. This suggests that FP presents in early psychosis alongside an overall more severe hallucination profile, rather than simply being an expression of paranoia. One limitation of this analysis is the relatively low scores for delusions across the sample; nevertheless, the association with emotional impact of hallucinations in the FP group is indicative of clinical and functional significance for people with psychosis. Delineating whether FP stands alone as a psychotic symptom, is an inference based on the experience of other symptoms, or reflects some kind of marker of multisensory hallucinatory experiences will be important to explore in future research.

The original study ran for a further two years with annual interviews of participants. At 12- and 24-month follow-ups, FP was less frequent as an overall proportion of participants. Within those who completed the second assessments (*n* = 23), FP dropped by half (61% at baseline to 30% at 12 months). For those who completed all three assessments (*n* = 15), FP as a proportion was observed in 60% of cases, then 27%, then 20%. This pattern of decline over time is consistent with other positive symptoms, although experiences may persist in a minority. In later interviews participants were in some cases able to offer more detailed descriptions of the experience, including the challenges of reporting it:

“I’ve found myself avoiding that question quite a few times like in therapy and things like that. It’s so … it is so difficult for me to describe that like sense. Because it feels like … it feels like no one else has ever experienced it, like I don’t know of anyone else, because like no … I don’t know … no one knows how to describe it!”

[Xander, 24-month assessment]

As noted by Critchley^14^, the elusive nature of FP raises the risk that such experiences are missed in the clinic and research despite their significance to the people who have them^3^. The data presented here show what is possible with an exploratory approach, but future identification will require the development of more systematic assessment methods. This could include multimedia tools similar to the Body Disturbances Inventory (BODI)^15^, which uses verbal and graphical descriptions to capture unusual bodily states. Experimental methods to induce presences have been successful in modelling FP in Parkinson’s^16,17^ and suggest that disruptions to bodily self-consciousness could underlie some experiences in psychosis; such accounts, however, do not explain the negative emotional impact FP appear to have. Exposure to adverse events has also been associated with FP in the general population, suggesting a role for trauma in its presentation^11^. Further research in larger clinical samples will be key to understanding the origins of distressing FP and opportunities for effective support and management.

### Methods & data availability

Forty people within their first nine months of using Early Intervention in Psychosis services were recruited for the original study^8^ across two large NHS trusts in Northern England (see Table 1). Participants had to be hearing voices at least once a week for a month and without a suspected duration of untreated psychosis over 5 years. All participants completed the HtV Phenomenological Interview and PSYRATS with a trained interviewer, along with a questionnaire battery and cognitive assessment (reported elsewhere^18^). The HtV interview was semi-structured and progressed from general to specific examples of unusual experiences, following participants’ choice of language wherever possible, bracketing out expectations of “typical” experiences and using follow-up questions to clarify how specific experiences differed from similar real or unusual experiences (see Supplementary Materials). The term “presence” was generally not used in questioning unless volunteered by participants; interviewer prompts could include follow-up questions like “Are they always in the same form (e.g. a voice, a vision, a presence?)”. Content and thematic analysis were used to derive the coding frame by an interdisciplinary team. Felt presences were coded from the data for “Experiences of feeling someone or something is present even when it cannot be seen or heard”; the code would also not be applied if there were clear examples of other hallucinations occurring concurrently. FP codes were then compared with a selection of a priori codes selected for theoretical relevance. Because of the exploratory nature of the research, associations between codes and group differences are presented here via log-transformed odds ratios and effect sizes (Cohen’s *d*). A full list of code definitions can be found in the original study^8^. All data for the above analyses are available to download from OSF.

## Supplementary information

Supplementary Materials
