# Supplementary material for: Experiences of felt presence in first episode psychosis
Source: Schizophrenia (Heidelb). 2025 Nov 26;11(1):143. doi: 10.1038/s41537-025-00690-2 (PMC12657941; doi:10.1038/s41537-025-00690-2)
Supplement: Supplementary file 1 — Supplementary Materials [file 41537_2025_690_MOESM1_ESM.docx]

**Supplementary Materials: Alderson-Day et al. (2025). Experiences of felt presence in first episode psychosis**

**The Hearing the Voice Phenomenology Interview (Alderson-Day, Woods et al., 2021)**

**Introductory comments**

“For this interview I’m going to be asking some questions about the different kinds of experiences you’ve been having recently. Quite a few of the questions are going to be about hearing voices that other people cannot hear. People sometimes worry about talking about this topic: Do you have any concerns? Is it OK to ask some questions about it?”

“If you would like to stop at any point, or would prefer to talk about something else, then it is absolutely fine to do so”

“Some of my questions are going to refer to “voices”, but people can mean lots of different things by that. For some people that might involve sound, while for others it might feel more like someone or something is communicating with you. Others describe their voices as a form of telepathy, or loud thoughts. Our aim is understand these experiences in terms that make sense for you, so please use whatever language you’re most comfortable with.”

**i. Initial voice description**

“Could you try to describe to me some of the voice (or voice-like) experiences you’ve been having?”

Prompts:

- *How, if at all, are these experiences different from your own thoughts?*
- *How, if at all, are these experiences different from hearing the voice of someone who is present in the room?*
- *Are there any other senses (e.g. images, tastes or smells) involved in the experience?*
- *Does it have a location (i.e. does it feel like you can tell where the voice is coming from)?*
- *Number of different voices/identities (if volunteered)*
- *If there is more than one voice, how do you distinguish between them? i.e. do they sound different, do they have different characters, do they say different things?*
- *When was the last time you had this kind of experience?*

**ii. General background**

“Could you tell us a bit about what life was like for you, and how you were feeling, when you first started having these experiences?”

Prompts:

- *Do you remember when you first heard a voice? (establish age estimate)*
- *If so, can you describe it?*
- *Was it similar to your recent voice/voice-like experiences?*
- *How were things going at the time?*
- *When did you first seek help or treatment?*

**iii. Emotions, feelings & anticipation**

“How does it feel when you have the experience?”

Prompts:

- *What kinds of moods or emotions are associated with your voices?*
- *Do you know when you are about to experience a voice? If so, how?*
- *Does your body feel different when you experience voices?*
- *Are there particular times or places when the experience is likely to occur?*
- *How do you feel towards your voice? Is this always the case, or does it vary?*

**iv. Communication & content**

“Do your experiences contain messages of any kind?”

Prompts:

- *What kinds of things does the voice say?*
- *Are there specific words or sentences that are used? Can you give me any examples?*
- *Do you always understand the message (what is being said)?*
- *How is it expressed? (e.g. friendly, unfriendly, angry, dominant, commanding, supportive, loud, quiet)*
- *How can you tell how it is expressed? Is it the tone of voice, for example?*
- *Is the message ever spiritual or religious?*

**v. Character & agency**

“*Does it feel as though the experiences have their own character or personality?*

Prompts:

- *Do you know who they are? If so, how?*
- *Do they remind you of anyone you know or have known?*
- *If so, is it the voice or what they say that reminds you of this person?*
- *Are they always in the same form (e.g. a voice, a vision, a presence?)*
- *Do they mean well?*
- *Do they know things that you do not?*
- *Are they a spiritual being?*

**vi. Change over time & life impact**

“Have your experiences changed at all since they first started?”

**Prompts**

- *Has it tended to happen more or less over time?*
- *Over time, have you been able to influence your voices? If so, how?*
- *How, if at all, have your voices affected your relations with other people?*

**vii. Personal meaning & interpretation**

“Why do you think these experiences are happening?”

Prompts:

- *What do the voices mean to you?*
- *Do you consider hearing voices to be a special ability or skill?*
- *Have they been caused by something that has happened to you, do you think?*
- *How do you think other people view this experience?*
- *Have you developed any ideas about why you have these experiences?*
- *Are you a religious or spiritual person?*
- *Have any of your family members had similar experiences?*

**viii. Other experiences**

“Is there anything else we haven’t talked about yet, but is an important part of your experience?”

Prompts:

- *Presence of any other senses or multisensory elements (if not already covered)*
- *Have you also had any other experiences that you think might be linked?*
